# Relationship between dairy product intake and sense of coherence among middle and high school students in Japan

**DOI:** 10.1371/journal.pone.0279232

**Published:** 2022-12-20

**Authors:** Kato Yoshiko, Kazumi Nagano, Chenghong Hu, Tomoyuki Furuyashiki

**Affiliations:** 1 Graduate School of Human Development and Environment, Kobe University, Kobe, Hyogo, Japan; 2 Kobe University Secondary School, Kobe, Hyogo, Japan; 3 Graduate School of Medicine, Kobe University, Kobe, Hyogo, Japan; 4 Japan Agency for Medical Research and Development, Chiyoda, Tokyo, Japan; Chiang Mai University, THAILAND

## Abstract

Despite the growing attention toward the effects of dairy intake on stress and mental health, its relationship to psychological constructs that affect mental health remains poorly understood. We conducted a cross-sectional study (Study 1) and a longitudinal study (Study 2) to examine the association between food intake and stress resilience in Japanese middle and high school students. In Study 1, 865 participants (412 males and 453 females) completed the questionnaires. In Study 2, 109 students (51 males and 58 females) participated each year from 2016 to 2018. Dietary intake was assessed using a brief self-administered diet history questionnaire. Stress resilience was evaluated using a 13-item sense of coherence (SOC) questionnaire. Correlation coefficients were calculated in Study 1 to investigate the relationship between food group intake and SOC. In Study 2, a cross-lagged panel model was tested using structural equation modeling to investigate the effect of dairy product consumption on SOC. Study 1 revealed that only dairy product intake positively correlated with SOC and other food intake indicated no significant relationship. Study 2 indicated that augmented dairy product intake was positively associated with SOC. Among all foods, only dairy products were associated with SOC in adolescents. Although the association was weak, the longitudinal study confirmed that dairy consumption was associated with SOC. Randomized controlled trials are necessary to examine the causal relationship.

## Introduction

Mental health and illness influence individual well-being and impose a large economic burden on the society [1, 2]. Many medical studies have long focused on pathophysiology of mental illnesses [3], contributing to treating mental illnesses through the development of novel drugs and other treatments. On the other hand, few studies have attempted to develop methods for preventing mental illnesses before onset and progression. A prophylactic approach to maintain and improve mental health and prevent mental illnesses is important, especially because mental illness frequently progresses over time [4].

Epidemiological studies have suggested an association between dairy food intake and mental health. Dairy consumption has been inversely correlated with depressive symptoms [5, 6]. Furthermore, oral administration of substances derived from milk proteins ameliorated chronic stress-induced depressive- and anxiety-like behaviors in rodents. These findings suggest that dairy product intake influences stress and mental health [7]. Other kinds of food have been effectively associated with stress and mental health [8–12]. In contrast, “junk” [13] and processed foods [8, 11] have been associated with poorer mental health. These findings suggest that different types of food are either beneficial or harmful to mental health. The relationship between dietary consumption and mental health might help researchers develop prophylactic dietary approaches for maintaining and improving mental health.

Despite the growing attention toward the effects of dietary intake (especially dairy products) on stress and mental health, potential psychological effects that might impact mental health remain poorly understood. Selye conceptualized stress as a strain of mental and physical functions caused by adverse and demanding conditions [14]. Basic and clinical studies have indicated that stress is an important risk factor for mental illnesses, especially depression and posttraumatic stress disorder [15]. The salutogenesis model of health describes internal and external resources that individuals use to cope with stress and foster well-being. Here sense of coherence (SOC) is conceptualized as an internal resource [16].

SOC refers to maintaining health—even under stressful situations—and structuring comprehensibility, manageability, and meaningfulness [17]. SOC may mediate the relationship between mental illnesses and various risk factors [18, 19]. Therefore, SOC is believed to help maintain and improve mental health, thereby preventing mental illnesses. Some studies reported a significant relationship between SOC and healthy dietary consumption [20, 21]. However, the relationships between dairy food consumption and SOC, well-being, and stress responses have not been studied.

This was the first cross-sectional study to explore the association between SOC and food group intake in Japanese middle and high school students, referring to the relationships among mental health indices, well-being, and stress responses. Using a longitudinal model, we subsequently investigated the relationship between dairy intake and SOC in Japanese middle school students.

## Study 1: Relationship among food intake, mental health, and soc

### Methods

#### Participants

Questionnaires were distributed to 931 middle and high school students in 2016; of them, 865 students (412 males and 453 females) completed the questionnaires. All participants were informed about the study and gave consent to participate. Protocols for Study 1 were approved by the Human Ethics Committee of the Graduate School of Human Development and Environment at Kobe University and by the Human Ethics Committee of Kobe University Secondary School. Informed consent was obtained from a parent and/or legal guardian according to the Kobe University Secondary School rules. The study procedures were carried out following the guidelines approved by the Human Ethics Committees.

### Measurements

*Background factors*. We collected data on participants’ sex, age, family size, body weight status, self-reported health status, and stage of change for healthy eating based on the transtheoretical model [22]. The body weight status was assessed using body mass index (BMI), calculated from self-reported heights and weights. The stage of change regarding healthy eating was assessed by asking participants to indicate which of the following eating habits best represented them: “precontemplation” for those who do not plan to start healthy eating (will not take action within 6 months); “contemplation” for those who contemplate healthy eating (intend to take action within 6 months); “preparation” for those who plan to change their eating habits soon and prepare for the action (intend to take action within next months); “action” for those who started healthy eating within 6 months; and “maintenance” for those who have been eating healthy for more than 6 months.

*Dietary assessment*. Dietary intake was estimated from a validated food frequency questionnaire and the brief self-administered diet history questionnaire (BDHQ) that measures dietary history over the preceding month. The BDHQ was developed based on a comprehensive version of a validated self-administered diet history questionnaire (DHQ) [23]. The BDHQ 15y is a revision of the adult version of the BDHQ; its validity had been previously confirmed [24]. We used the BDHQ 15y, which lists 67 food items for older children and adolescents, considering their legibility and knowledge of food. We estimated the intake of 14 foods using a computer algorithm for the BDHQ 15y.

*Sense of coherence*. SOC was measured using a 13-item SOC questionnaire. This scale is a shortened version of a 29-item SOC questionnaire [16] and was translated into Japanese [25]. Participants recorded their current feelings according to a seven-point Likert scale.

*Stress response*. The children’s stress response (CSR) test is a self-completed questionnaire to measure stressful situations for elementary, middle, and high school students in Japan [26]. This scale is composed of three subscales (12 items in total): “irritability,” “lethargy,” and “depression.” CSR asks the respondent about his or her emotions during the preceding week, corresponding to each of the three subscales. Participants answered each item on a four-point Likert scale.

*Wellbeing (IKIGAI)*. Well-being was estimated using the Ikigai scale [27]. “Ikigai” is a Japanese word meaning “worth living” and indicates well-being in Japanese. The scale comprises nine items that are divided into three subscales: “personal growth,” “life satisfaction,” and “social role.” Participants recorded their daily feelings toward each item on a five-point Likert scale.

### Statistical analyses

Correlation coefficients were calculated to examine the relationship between food group intake and SOC, an index of stress resilience. At the same time, stress response and well-being related to stress resilience were also examined how related to food group intake by calculating correlation coefficients.

Before the primary analyses were performed, Cronbach’s alpha values were calculated to determine the reliability of SOC, stress response, and well-being measures. Food intake was highly influenced by BMI, age, and educational level [28]. The educational level was homogenized in this study. Sex-based differences in food consumption, SOC, stress response, and well-being were evaluated by the U-test as the data was not evenly distributed. Since sex and BMI affect food consumption, the correlation analysis to explore the relationship among food group intake, SOC, stress response, and well-being were adjusted by BMI and sex. All statistical analyses were performed using SPSS Version 23 software.

## Results

The demographic profiles of the participants are provided in Table 1. The mean ages of the males and females were 14.7 (SD 1.7) and 14.6 (SD 1.6) years, respectively. The mean BMI was 19.4 (SD 2.5) kg/m^2^.

**Table 1:** Participants’ characteristics (*n* = 865).

		Male		Female	
		*N*	%	*N*	%
Age	12 y	47	1.5	47	10.9
	13 y	71	18.6	85	18.0
	14 y	85	19.7	97	21.0
	15 y	65	16.6	79	16.6
	16 y	79	2.2	85	19.0
	17 y	47	1.0	47	10.9
	18 y	18	0.5	13	3.6
Family	2 people	6	1.5	7	1.5
	3 people	76	18.4	103	22.7
	4 people	244	18.4	256	56.5
	5 people	68	16.5	73	16.1
	6 people	9	2.2	10	2.2
	7 people	4	1.0	2	0.4
	8 people	2	0.5	0	0.0
	N/A	3	0.7	2	0.4
Health status	Best	43	10.4	26	5.7
	Very good	61	14.8	96	21.2
	Good	206	50.0	213	47.0
	Normal	68	16.5	90	19.9
	Not good	20	4.9	15	3.3
	Not very good	6	1.5	5	1.1
	N/A	8	1.9	8	1.8
Stages of change for healthy eating	Precontemplation	102	32.3	89	25.3
	Contemplation	51	16.1	70	19.9
	Preparation	51	16.1	59	16.8
	Action	30	9.5	47	13.4
	Maintenance	82	25.9	87	24.7
	N/A	96	23.3	101	22.3

Analysis of food group intake, SOC, stress response, and well-being by sex are shown in Table 2. Cronbach’s alpha values for measuring SOC, stress response, and well-being ranged from 0.72 to 0.81. The male participants consumed more grains, beans, fish and shellfish, meats, eggs, dairy products, fats and oils, and nonalcoholic beverages than female participants. The depression score was higher in females, and the social role score was higher in males. The effect size for the grain was r = 0.47. This implies that there may be a disparity in sex in grain intake. However, the effect sizes for the other foods, SOC, stress response, and well-being were minimal. The sex differences in other foods, SOC, stress response, and well-being were considered minor.

**Table 2:** Means, standard deviations, and medians for variables and the results of the Mann–Whitney U-test by sex.

		Total (*n* = 865)			Male (*n* = 412)			Female (*n* = 453)			*r (*Effect size*)*	*p-value*
		M	SD	MED	M	SD	MED	M	SD	MED		
Food group (g/day)	Grains	513.8	339.7	433.0	644.2	376.5	579.7	395.2	249.2	364.6	.47	< .001
	Potatoes	36.5	33.2	31.0	37.5	35.1	31.6	35.6	31.3	31.0	.05	.145
	Sugar and sweetener	2.5	1.6	2.2	2.4	1.6	2.2	2.5	1.6	2.2	.02	.655
	Beans	50.8	43.7	39.4	53.6	44.4	39.4	48.2	42.9	36.2	.09	.011
	Green and yellow vegetables	123.3	83.9	104.7	120.5	85.4	104.5	125.9	82.5	106.9	.05	.188
	Other vegetables	157.8	101.1	134.3	153.1	104.4	127.7	162.1	98.0	140.7	.06	.066
	Fruits	165.2	171.4	117.4	169.3	182.3	117.4	161.5	161.0	116.6	.00	.964
	Fish and shellfish	73.4	57.0	58.5	83.3	65.5	65.2	64.5	46.2	51.9	.16	< .001
	Meats	89.0	55.5	76.0	96.5	60.2	79.1	82.3	49.9	69.7	.15	< .001
	Eggs	50.4	33.4	50.7	49.2	34.9	50.7	51.5	31.9	46.5	.07	.041
	Dairy products	359.2	309.6	265.5	443.0	357.0	336.7	282.9	234.6	231.8	.24	< .001
	Fats and oils	16.1	8.1	14.8	17.1	8.4	16.3	15.2	7.8	13.8	.13	< .001
	Confections	73.7	64.4	56.4	72.2	66.5	52.8	75.1	62.6	60.0	.05	.174
	Nonalcoholic beverages	765.9	448.8	728.3	831.8	498.2	795.9	706.0	389.6	688.5	.13	< .001
SOC		4.0	0.9	4.0	4.0	0.9	4.0	4.0	0.9	4.0	.01	.794
Stress response	Irritability	2.4	0.8	2.3	2.3	0.8	2.3	2.4	0.8	2.3	.02	.625
	Lethargy	2.6	0.8	2.5	2.6	0.8	2.5	2.6	0.8	2.5	.02	.480
	Depression	2.0	0.7	2.0	1.9	0.7	1.8	2.1	0.7	2.0	.09	.010
Well-being	Life satisfaction	3.4	0.9	3.3	3.3	0.9	3.3	3.5	0.9	3.3	.06	.071
	Personal growth	3.9	0.9	4.0	3.8	0.9	4.0	3.9	0.9	4.0	.05	.120
	Social role	2.8	1.0	3.0	2.9	1.0	3.0	2.8	0.9	2.7	.09	.011

M; Mean value, SD; Standard Deviation, MED; Median

Correlations among food group intake, SOC, stress response, and well-being are shown in Table 3. SOC was significantly and positively correlated with dairy products (*r* = 0.091, *p* < 0.01). SOC was not correlated with the other foods. For stress response, the “irritability” score was positively correlated with potatoes (*r* = 0.115, *p* < 0.01), confections (*r* = 0.158, *p* < 0.001), and nonalcoholic beverages (*r* = 0.067, *p* < 0.05). The “lethargy” score was positively correlated with confections (*r* = 0.112, *p* < 0.01). The “depression” score was positively correlated with green and yellow vegetables (*r* = 0.092, *p* < 0.01) and confections (*r* = 0.107, *p* < 0.01). For well-being, the “life satisfaction” score was positively correlated with fish and shellfish (*r* = 0.102, *p* < 0.01) and dairy products (*r* = 0.067, *p* < 0.05). The “personal growth” score was positively correlated with sugar and sweetener (*r* = 0.085, *p* < 0.05), beans (*r* = 0.101, *p* < 0.01), other vegetables (*r* = 0.085, *p* < 0.05), fish and shellfish (*r* = 0.076, *p* < 0.05), eggs (*r* = 0.091, *p* < 0.01), and dairy products (*r* = 0.093, *p* < 0.01). The “social role” score was positively correlated with grains (*r* = 0.081, *p* < 0.05), sugar and sweetener (*r* = 0.102, *p* < 0.01), beans (*r* = 0.078, *p* < 0.05), fish and shellfish (*r* = 0.155, *p* < 0.001), meats (*r* = 0.071, *p* < 0.05), eggs (*r* = 0.079, *p* < 0.05), and dairy products (*r* = 0.092, *p* < 0.01). SOC was negatively correlated to the stress response (*r* = −0.523 to −0.471, *p* < 0.001) and positively correlated to well-being (*r* = 0.293 to 0.543, *p* < 0.001).

**Table 3:** Correlations among food group intake and SOC, stress response, and well-being.

				Irritability		Lethargy		Depression		Life satisfaction		Personal growth		Social role	
Food group															
	Grains	.014		.007		−.043		.003		.016		.017		.081	*
	Potatoes	−.044		.115	**	.012		.055		−.026		−.009		.016	
	Sugar and sweetener	.041		−.058		−.040		.003		.065		.085	*	.102	**
	Beans	.004		.009		−.042		.049		.052		.101	**	.078	*
	Green and yellow vegetables	−.033		.061		.023		.092	**	.018		.051		.022	
	Other vegetables	.001		.004		−.011		.065		.057		.085	*	.040	
	Fruits	.031		.055		−.025		.028		.037		.019		.044	
	Fish and shellfish	.054		.005		−.005		.054		.102	**	.076	*	.155	***
	Meats	−.029		.031		−.022		.037		.060		.059		.071	*
	Eggs	−.025		.014		.010		.033		.045		.091	**	.079	*
	Dairy products	.091	**	−.042		−.031		−.042		.067	*	.093	**	.092	**
	Fats and oils	−.027		.055		.024		.061		.024		.037		.057	
	Confections	−.053		.158	***	.112	**	.107	**	−.002		.007		.045	
	Nonalcoholic beverages	.004		.067	*	.046		.051		−.010		.029		.010	
SOC		—		−.471	***	−.523	***	−.510	***	.543	***	.293	***	.436	***

***p < .001

**p < .01; and *p < .05; adjusted variables are sex and BMI.

## Study 2: Longitudinal study of dairy product intake effects on soc among japanese middle and high school students

### Methods

A questionnaire survey was conducted once every year from 2016 to 2018. The survey population was all students (*n* = 118) enrolled in the collaborating middle school for three years, from 2016 to 2018. A total of 109 responses (51 males and 58 females) were analyzed, excluding those whose answers were incomplete.

Data were collected during approximately the same month (i.e., June) every year using the same self-reported questionnaire. The questionnaire collected data on the background characteristics, SOC, and dietary intake, as previously described for Study 1. The participants were informed about the aim of this study and answered the questionnaires after giving their consent. Study 2 was approved by the Human Ethics Committee of the Graduate School of Human Development and Environment at Kobe University and by the Human Ethics Committee of Kobe University Secondary School. According to the Kobe University Secondary School rules, informed consent was obtained from a parent and/or legal guardian according to the Kobe University Secondary School rules. The study procedures were carried out following the guidelines approved by the Human Ethics Committees.

### Model

**Fig 1:**
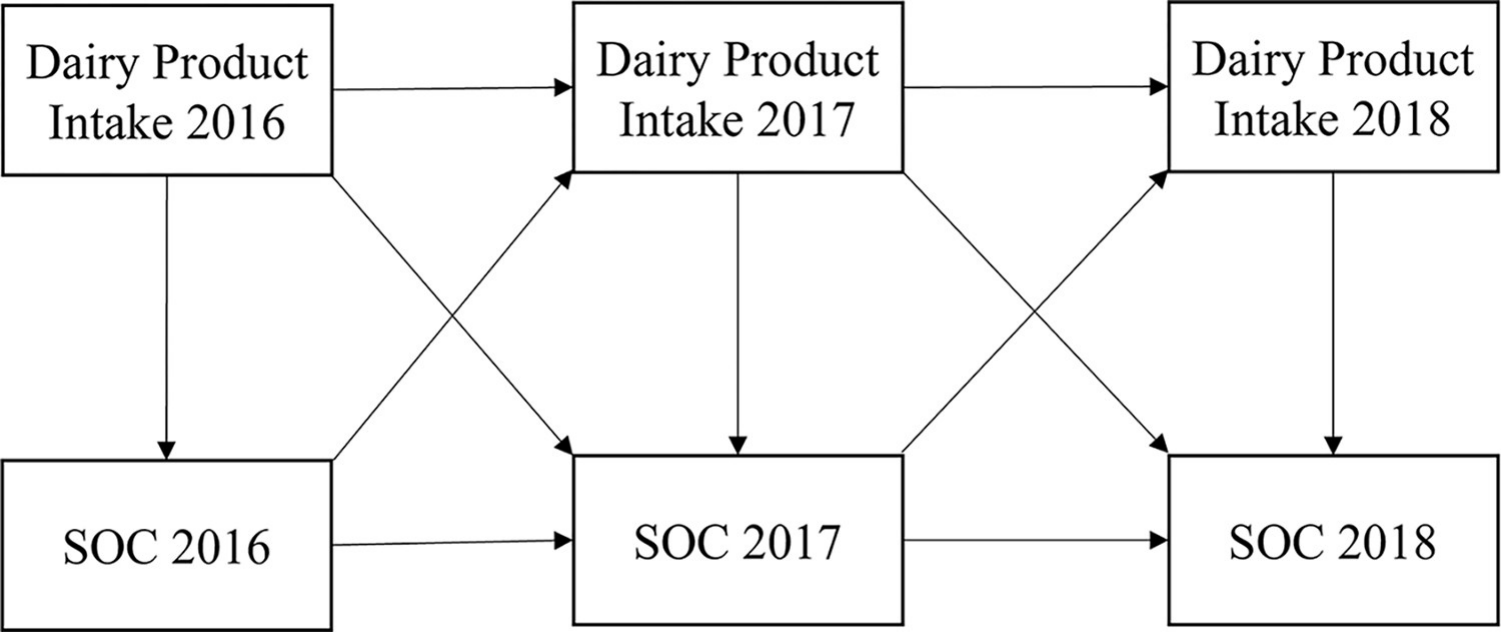
Conceptional model of the association between SOC and dairy product intake.

This study was designed to longitudinally examine the effects of dairy product intake on SOC. Referring to related works [29, 30], we developed a cross-lagged structural equation model to explain the relationship between dairy product intake and SOC (Fig 1). In this model, dairy product intake was defined as the estimated sum of ice cream, yogurt, cheese, low-fat milk, full-fat milk, and lactic acid bacteria beverages (LABB) based on the BDHQ 15y.

### Statistical analyses

The cross-lagged panel model described above was tested using structural equation modeling to address the influence of dairy product intake on SOC. First, this model was adjusted for sex and BMI (sex and BMI confounding model; SB model). Second, the correlations between the consumption of dairy products and other foods were examined, and we identified foods related to dairy product consumption. Based on the results, sex, BMI, and identified foods were used as the confounding variables (sex, BMI, and food consumption confounding model; SBF model). The following indicators of model fit statistics were provided to examine the data adaptation for the model: root mean square error of approximation (RMSEA), comparative fit index (CFI), the goodness of fit index (GFI), adjusted goodness of fit index (AGFI) and Akaike information criterion (AIC). All statistical analyses were conducted with SPSS Version 23 and AMOS Version 23.

## Results

At the time of the first survey in 2016, all students were first grade in junior high school, and their mean age was 12.2 years (SD 0.4) for the males and 12.3 years (SD 0.5) for the females. The mean BMI values were 17.3 (SD 2.3) kg/m^2^ in 2016, 17.9 (SD 1.6) kg/m^2^ in 2017, and 18.8 (SD 2.6) kg/m^2^ in 2018 for the males and 17.8 (SD 2.2) kg/m^2^ in 2016, 18.4 (SD 1.9) kg/m^2^ in 2017, and 19.1 (SD 2.0) kg/m^2^ in 2018 for the females.

Table 4 shows the mean dairy product intake according to sex. The results showed that males consumed significantly more total dairy products than females during all three years.

**Table 4:** Means, standard deviations, and medians for dairy product intake and the results of the *U*-test by sex.

Food group (g/day)		Total (*n* = 109)			Males (*n* = 51)			Females (*n* = 58)					*r* (Effect size)	*p-value*
		M	SD	MED	M	SD	MED	M		SD		MED		
**2016 (1**^**st**^ **grade)**														
	Ice cream	48.8	41.4	46.5	47.9	43.3	50.7	49.5		40.0		46.5	.09	.372
	Yogurt	121.5	114.4	76.0	157.0	119.8	106.4	90.3		100.5		34.9	.41	< .001
	Cheese	16.2	22.9	5.1	18.7	25.6	12.7	14.0		20.2		4.6	.24	.014
	Low-fat milk	67.3	124.3	0.0	92.1	153.4	11.8	45.4		87.2		0.0	.18	.067
	Full-fat milk	147.3	168.8	116.2	181.7	176.2	177.3	117.0		157.4		58.1	.37	< .001
	LABB	32.6	64.1	6.5	47.2	80.9	15.2	19.7		41.0		6.5	.22	.020
	Total	433.6	291.1	379.5	544.6	306.9	506.6	335.9		239.2		269.1	.39	< .001
**2017 (2**^**nd**^ **grade)**														
	Ice cream	42.9	50.7	20.3	44.9	54.2	20.3	41.1		47.7		18.6	.12	.217
	Yogurt	116.8	114.4	76.0	140.3	119.3	106.4	96.1		106.6		52.3	.31	.001
	Cheese	18.5	20.4	11.6	18.5	21.4	12.7	18.5		19.6		11.6	.07	.471
	Low-fat milk	74.3	158.2	0.0	101.8	203.6	0.0	50.2		99.0		0.0	.08	.401
	Full-fat milk	169.3	195.8	116.2	229.4	228.1	177.3	116.4		144.7		58.1	.36	< .001
	LABB	23.2	36.3	6.5	31.1	40.4	7.1	16.2		31.0		6.5	.25	.010
	Total	444.9	339.2	384.2	566.0	390.5	488.9	338.5	244.1		258.6		.34	< .001
**2018 (3**^**rd**^ **grade)**														
	Ice cream	43.3	46.0	20.3	45.2	50.7	50.7	41.7	41.9		18.6		.17	.071
	Yogurt	100.9	105.9	69.7	110.2	111.1	76.0	92.7	101.3		34.9		.21	.028
	Cheese	14.6	17.4	11.6	15.0	21.2	5.1	14.2	13.4		11.6		.03	.788
	Low-fat milk	39.0	100.1	0.0	56.0	126.1	0.0	24.0	67.5		0.0		.19	.048
	Full-fat milk	125.7	168.0	63.3	186.5	194.8	177.3	72.2	118.3		10.8		.42	< .001
	LABB	20.6	32.8	6.5	25.0	38.1	7.1	16.7	27.1		6.5		.09	.358
	Total	198.6	31.2	172.8	231.5	3.8	233.6	169.7	4.1		172.8		.35	< .001

M; Mean value, SD; Standard Deviation, MED; Median

LABB: lactic acid bacteria beverages.

Regarding specific food items, the intake of full-fat milk was significantly higher in males than in females during all three years. Yogurt and LABB intakes were significantly higher in males than in females only in 2016. During the three years, males consumed more full-fat milk and dairy products than females.

**Fig 2:**
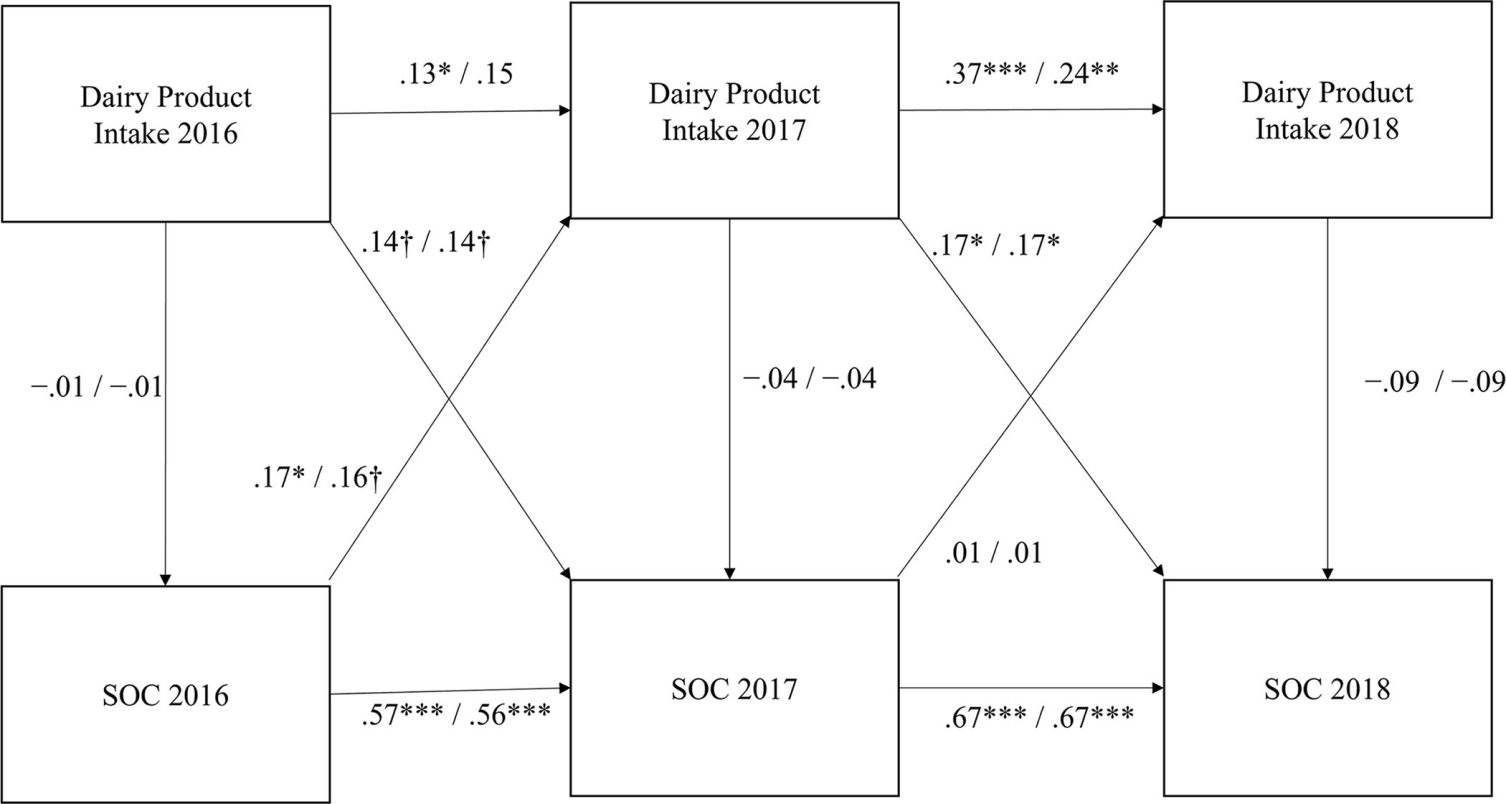
Structural model of the associations between dairy product intake and SOC. ^**†**^*p* < .1; *p < .05; **p < .01; and ***p < .001. The coefficients shown are standardized path coefficients. Above: SB model/adjusted variables are sex and BMI. Below: SBF model/adjusted variables are sex, BMI, the consumption of green and yellow vegetables, fruits, and fish and shellfish in 2016, the consumption of grains, sugar and sweetener, beans, fruits, fish and shellfish, and meats in 2017, and the consumption of grains, fruits, fish and shellfish, fats and oils, and confections in 2018.

As shown in Fig 2, our cross-lagged panel model for SOC and dairy product intake provides an acceptable fit to the data according to the following values for the SB model (GFI = 0.95, AGFI = 0.88, CFI = 0.98, RMSEA = 0.06, and AIC = 95.14) and the SBF model (GFI = 0.87, AGFI = 0.78, CFI = 0.98, RMSEA = 0.03, and AIC = 436.66).

BMI and sex were adjusted in the SB model. In the SBF model, sex, BMI, and food consumption, which are associated with dairy food intake, were therefore adjusted. The adjusted variables were the consumption of green and yellow vegetables, fruits, and fish and shellfish in 2016, the consumption of grains, sugar and sweetener, beans, fruits, fish and shellfish, and meats in 2017, and the consumption of grains, fruits, fish and shellfish, fats and oils, and confections in 2018. In 2016, the consumption of green and yellow vegetables (*r* = 0.27, *p* < 0.01), fruits (*r* = 0.32, *p* < 0.01), and fish and shellfish (*r* = 0.23, *p* < 0.05) was associated with dairy product intake. In 2017, the consumption of grains (*r* = 0.23, *p* < 0.05), sugar and sweetener (*r* = 0.22, *p* < 0.05), beans (*r* = 0.26, *p* < 0.01), fruits (*r* = 0.21, *p* < 0.05), fish and shellfish (*r* = 0.26, *p* < 0.01), and meats (*r* = 0.22, *p* < 0.05) was associated with the dairy product intake. In 2018, the consumption of grains (*r* = 0.26, *p* < 0.01), fruits (*r* = 0.21, *p* < 0.05), fish and shellfish (*r* = 0.26, *p* < 0.01), fats and oils (*r* = 0.33, *p* < 0.001), and confections (*r* = 0.20, *p* < 0.05) was associated with the dairy product intake. No other foods were significantly associated with dairy product consumption.

Dairy consumption in 2016 was associated with SOC in 2017 (*β* = 0.14, *p* = 0.089/*β* = 0.14, *p* = 0.081), although not significantly. The direct effect from dairy intake in 2016 to SOC in 2017 was 0.14/0.14, and the indirect effect through SOC in 2016 was −0.01/−0.01. However, dairy consumption in 2017 was significantly associated with SOC in 2018 (*β* = 0.17 *p* = 0.030/*β* = 0.17 *p* = 0.026). The direct effect was 0.17/0.17, and the indirect effect was –0.06/–0.05.

SOC in 2016 was associated with dairy intake in 2017 in the SB model (*β* = 0.17, *p* = 0.048), although it was not associated with the SBF model (*β* = 0.16, *p* = 0.051). SOC in 2017 was not associated with dairy intake in 2018 (*β* = 0.01 *p* = 0.93/*β* = 0.01 *p* = 0.85).

## Discussion

We examined the association between intake of various food groups and psychological constructs related to the salutogenesis of mental health, namely SOC and well-being. Our results might be useful for developing a strategy for dietary interventions that promote mental health and prevent mental illnesses. Only dairy product intake was associated with SOC and all three dimensions of well-being. However, since the association was weak, we conducted a longitudinal study for three years to further investigate this relationship. The results again demonstrated that dairy consumption was weakly associated with SOC.

Dairy intake is inversely related to the trait “anger” in female students [31]. Low-fat dairy intake was associated with social functioning, reduced stress, and memory improvement. In contrast, whole-fat dairy, including ice cream and fat cream, was associated with poorer psychological well-being in middle-aged Australians [32]. However, these studies did not analyze psychological constructs related to stress resilience. One report indicated that low SOC scores were associated with fewer health-promoting dietary preferences [21]. Our findings provide the first evidence of an association between dairy product intake and SOC.

Most notably, we performed longitudinal analyses of the same participants over the three years, allowing us to develop a cross-lagged panel model to decipher the effect of preceding dairy intake on subsequent SOC scores after one year. SB model outperforms the SBF model according to the AIC value. Therefore, we examined the SB model’s goodness of fit. Most of the parameters are within recommended ranges [33, 34]. All the parameters of the structural model are within acceptable limits, except for the AGFI (0.88) being below the recommended 0.90. Most fit indices, especially GFI and AGFI, are reported to be impacted by sample size. A larger sample could produce higher fit indices [35, 36]. Given the relatively modest size of the sample, the SB model fits the data well.

Consequently, our findings in this study are the first to demonstrate the relationship between dairy food intake and stress resilience, compared to other studies with similar types of analyses, thus suggesting the causal role of dairy product intake on stress resilience and mental health. Dietary habits are often linked with other daily habits, perhaps due to educational environments; however, our study participants went to the same school and thus had similar educational backgrounds. We believe this design helps to isolate the effects of dietary intake from potential confounders. However, knowing about effective food intake for SOC through education could also promote healthy eating habits. It should be noted that all of our analyses were of adolescents (i.e., 12–18 years); therefore, our findings may not generalize to adults or other populations.

Increased dairy consumption was positively associated with the internal resource (SOC) to manage stress and foster well-being. It should be noted that SOC and stress response were assessed during the survey and the previous week, respectively, whereas BDHQ was used to assess dietary history over the previous month. Although the measurement period dichotomy could be a limitation of this study, BDHQ reportedly yields a consistent result with semi-weighed dietary records (DR) over the past four days, which is considered the most accurate estimate of food consumption so far [37]. Therefore, BDHQ can be reasonably compared to recent measurements of stress response and SOC. In any case, dietary intervention trials are required to establish the causal role of dairy consumption in improving mental health.

In addition to the internal resource, Antonovsky also investigated external resources for stress coping [16]. Social support is one such external resource. Ager et al. reported that perceived social support and team cohesion promote mental health [38]. Thus, another limitation of this study is that we did not examine the influence of social support. One cannot always obtain or perceive adequate social support; however, one can improve and maintain dietary habits alone. In other words, if improved dietary habits are separate from social support and help promote SOC, then SOC can be enhanced through individual efforts to improve dietary habits. Indeed, Suzuki et al. found an association between job strain and depressive symptoms and the protective impact of a balanced Japanese diet against depressive symptoms, especially in participants with active strain and low support [12]. Whether social support and healthy eating habits may cooperate in promoting SOC requires further study.

Finally, we cannot exclude the possibility of residual confounding factors due to unmeasured or imprecisely measured factors. In general, as people eat various foods at each meal, there may be correlations between dairy intake and other dietary items. However, the effect of dairy intake on SOC did not substantially attenuate after adjusting for intake of other foods related to dairy consumption. This provides a rebuttal to the interpretation that we found a spurious association that is simply a by-product of a healthy diet.

The rate of depression has been increasing dramatically in Japan [39]. Adolescents also face difficulties and suffer mental problems. Simultaneously, lifestyle disturbances (skipping breakfast, sleeping problems, gaming, etc.) are increasingly noticeable. In adolescence, stress affects the trajectory of brain development and is a strong risk factor for mental illnesses, including depression [40, 41]. Our findings imply that maintaining healthy lifestyle habits, such as eating healthy foods, is a valuable strategy to improve physical and mental health in adolescents. This study found the association between dairy product intake and SOC, but their causal relationship remains elusive. Hence, a double-blind, placebo-controlled study is warranted. However, it will be challenging to develop a dairy product placebo without identifying critical ingredients of the dairy product that augment the SOC. Moreover, evidence concerning how food intake impacts mental health in children is still limited. Recent studies have suggested that the gut microbiome and its metabolites affect animal behaviors and human mental states [42]. Multiple mechanisms—including leaky gut barrier, mucosal immunity, and autonomic nerves—likely mediate the gut–brain crosstalk. Unfortunately, their relationship with food components remains poorly understood. In the future, we will require a deeper understanding of the impact of dairy products on psychological and brain functions. This information may help consolidate the causal role of dairy products on mental health.

In conclusion, dairy consumption has a weak impact on stress resilience and well-being in adolescents. Additional dietary intervention trials will be required to establish any causal relationship.
